# Spin–orbit torque driven magnetization switching in W/CoFeB/MgO-based type-Y three terminal magnetic tunnel junctions

**DOI:** 10.1038/s41598-021-95422-8

**Published:** 2021-08-17

**Authors:** Shinji Isogami, Yohei Shiokawa, Atsushi Tsumita, Eiji Komura, Yugo Ishitani, Kosuke Hamanaka, Tomohiro Taniguchi, Seiji Mitani, Tomoyuki Sasaki, Masamitsu Hayashi

**Affiliations:** 1 National Institute for Materials Science, Tsukuba, 305-0047 Japan; 2 TDK Corporation, Chiba, 272-8588 Japan; 3 Research Center for Emerging Computing Technologies, National Institute of Advanced Industrial Science and Technology, Tsukuba, 305-8560 Japan; 4 The University of Tokyo, Tokyo, 113-8654 Japan

**Keywords:** Electrical and electronic engineering, Magnetic devices

## Abstract

We have studied current induced magnetization switching in W/CoFeB/MgO based three terminal magnetic tunnel junctions. The switching driven by spin—orbit torque (SOT) is evaluated in the so-called type-Y structure, in which the magnetic easy-axis of the CoFeB layer lies in the film plane and is orthogonal to the current flow. The effective spin Hall angle estimated from the bias field dependence of critical current (*I*_c_) is ~ 0.07. The field and current dependence of the switching probability are studied. The field and DC current induced switching can be described using a model based on thermally assisted magnetization switching. In contrast, the 50 ns long pulse current dependence of the switching probability shows significant deviation from the model, even if contribution from the field-like torque is included. The deviation is particularly evident when the threshold switching current is larger. These results show that conventional thermally assisted magnetization switching model cannot be used to describe SOT induced switching using short current pulses.

## Introduction

The spin–orbit torque (SOT) magnetoresistive random access memory (MRAM) is one of the emerging technologies for next generation memory devices^1, 2^. In SOT-MRAM, current is passed along a channel made of a non-magnetic heavy metal (HM) layer on which a magnetic tunnel junction (MTJ) is placed. The current passed along the HM layer generates spin current via the spin Hall effect that diffuses into the ferromagnetic free layer of the MTJ. Such spin current exerts SOT^3–5^ on the free layer magnetization. With appropriate condition, the SOT can induce magnetization switching depending on the direction to which the current flows within the channel. As the current needed to write information (i.e. switch the magnetization) does not flow across the tunnel barrier, the three terminal SOT-MRAM is considered to possess larger endurance compared to the conventional two terminal STT-MRAM^6^.

The three terminal SOT-MRAM can be categorized into three types^7^ depending on the geometry of the MTJs and the SOT channel: type-X, Y, Z refers to, respectively, magnetic easy-axis of the free layer of the MTJ pointing along the current flow direction (type-X), orthogonal to the current flow direction but lies in the film plane (type-Y), and perpendicular to the film plane (type-Z). It is now well understood that the switching dynamics of the three types are different. While types-X and Z allow sub-nanosecond switching of the magnetization^7–9^, type-Y requires nanoseconds long incubation time to cause the switching^4, 10, 11^. On the other hand, magnetization can be controlled in type-Y^12^ without any magnetic field, whereas a dc bias magnetic field is needed during the current application process for types-X and Z. The switching scheme of type-Y is close to that of conventional STT-MRAM. As field-free switching schemes for types-X and Z devices are currently being developed, the most straightforward approach to replace STT-MRAM with SOT-MRAM is to use the type-Y device.

Here we study SOT induced magnetization switching probability of type-Y three terminal MTJ. We use tungsten (W) as the channel material and CoFeB/MgO/CoFeB as the base element of the MTJ. The switching probability with DC current and pulse current are investigated and compared to model calculations based on thermally assisted magnetization switching. We include the field-like torque in the model in an attempt to account for the experimental results.

## Results and discussion

### Device structure and magnetization switching by field and current

**Figure 1:**
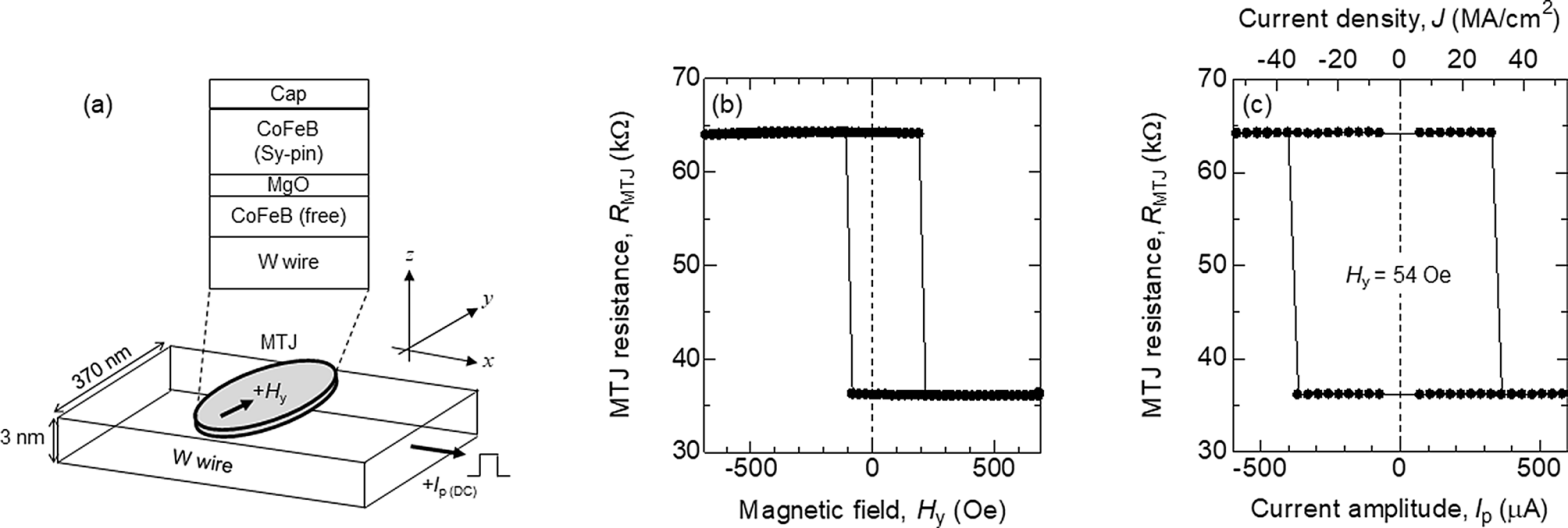
(**a**) Schematic illustration of type-Y three terminal magnetic tunnel junction (MTJ) including the film stacking. The coordinate axis is depicted together. (**b**) *H*_*y*_ dependence of the MTJ resistance *R*_MTJ_ at room temperature. (**c**) $R_{\rm{MTJ}}$ plotted as a function of the current pulse amplitude measured while a bias field of *H*_*y*_ ~ 54 Oe is applied. The duration of current pulse is ~ 50 ns.

Figure 1a shows schematic illustration of the three terminal MTJ^13^ consisting of a W channel^10, 14–16^ and the elliptical MTJ pillar. The MTJ consists of MgO barrier sandwiched by a CoFeB free layer and a CoFeB-based synthetic-antiferromagnet (SAF) pinned layer. The thickness of the free layer is 2 nm. The coordinate axis is sketched in Fig. 1a. The short and long axes of the elliptical MTJ pillar are 120 nm and 370 nm, respectively. The long axis of the pillar, which corresponds to the magnetic easy-axis of the free layer due to shape anisotropy, is parallel to the $y$-axis. The pinned layer magnetization points along ${+y}$. A variable amplitude pulse current (*I*_p_) with duration (${t}_{\rm{p}}$) fixed to 50 ns or DC current (*I*_DC_) (duration is ~ 1 s) is applied to the W channel to induce magnetization switching of the free layer via SOT. The pulse and DC current sources are different. Note that the rise and fall time of the DC current is significantly larger than those of the pulse current but are sufficiently smaller than the duration of the current (~ 1 s). Current passed along the W channel is parallel to the $x$-axis: positive current is defined as current flowing along $+x$. The geometry of the device used here is referred to as type-Y. To probe the magnetic state of the MTJ free layer, a DC bias voltage of 10 mV is applied to the MTJ. An external field *H*_*y*_ is applied along the $y$-axis to reset the magnetic state of the free layer as well as to apply a bias field during the channel current application. We have studied more than 15 devices with nominally the same device structure. Representative results from one device are shown: similar data are obtained for the other devices. All measurements are performed at room temperature.

Figure 1b shows the minor loop of the MTJ resistance (*R*_MTJ_) vs. *H*_*y*_. The high (~ 64 kΩ) and low (~ 36 kΩ) resistance states correspond to antiparallel (AP) and parallel (P) alignment of the free and pinned layers’ magnetization. The tunnel magnetoresistance (TMR) ratio and the resistance-area product are ~ 78% and ~ 1600 Ωμm^2^, respectively. As *H*_*y*_ is swept, switching from P to AP (AP to P) states are found at *H*_*y*_ ~ $-$ 100 Oe (~ 210 Oe). The center of the minor loop is shifted to + *H*_*y*_, which is due to the stray field from the pinned layer and/or the orange peel coupling of the free and pinned layers. The shift field, defined as *H*_s_, is ~ 54 Oe.

Figure 1c shows the *R*_MTJ_*-I*_p_ loop measured with a constant bias field *H*_*y*_ ~ 54 Oe. We pass a current pulse (50 ns long) through the W channel and measure the MTJ resistance while *H*_*y*_ is applied. The amplitude of the current pulse is varied from *I*_p_ =  + 550 μA to $-$ 550 μA and then reversed. The SOT switching from P to AP (AP to P) is observed at *I*_p_ ~ 390 μA (~ $-$380 μA). The change in the MTJ resistance *R*_MTJ_ is consistent with the *R* difference of the P and AP magnetic states. We define ${I}_{\mathrm{c}}^{\mathrm{P}(\mathrm{AP})}$ as the switching current when the initial state is the P (AP) state.

### Evaluation of the effective spin Hall angle

**Figure 2:**
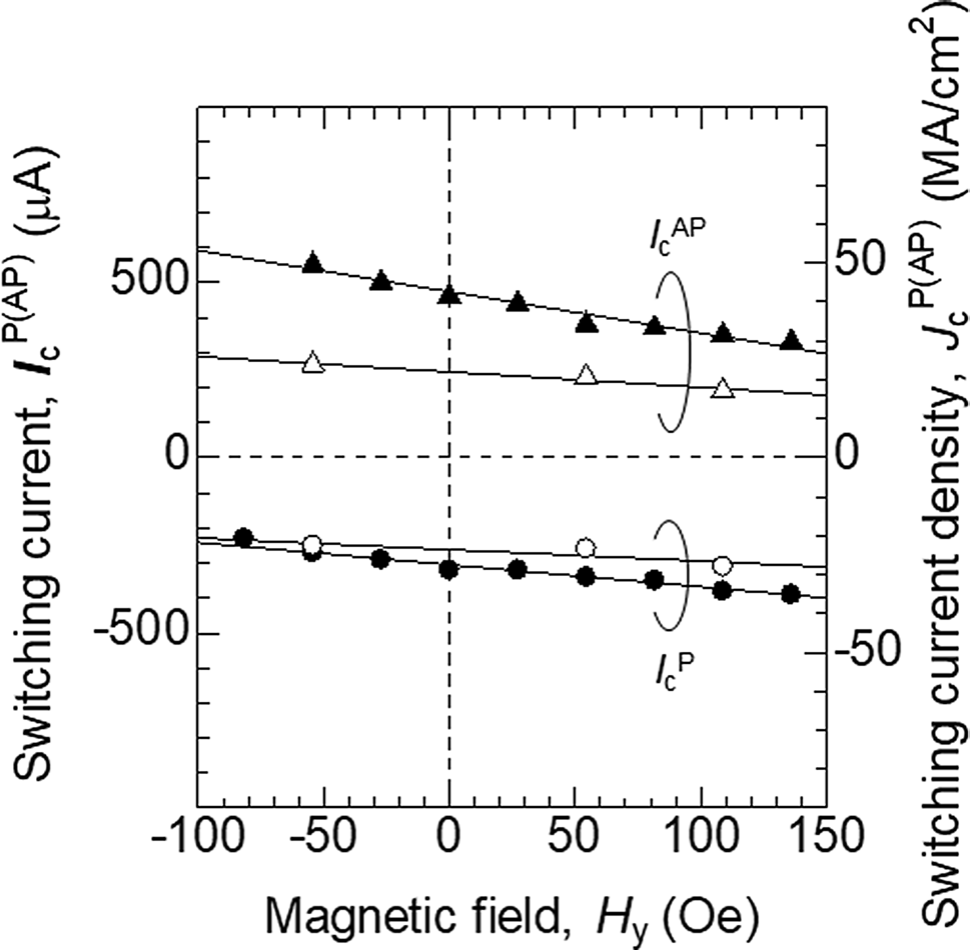
*H*_*y*_ dependence of the switching current ${I}_{\mathrm{c}}^{\mathrm{P}(\mathrm{AP})}$. The circles and triangles represent the switching from parallel (P) to antiparallel (AP) states (${I}_{\mathrm{c}}^{\mathrm{P}}$) and from AP to P states (${I}_{\mathrm{c}}^{\mathrm{AP}}$), respectively. The solid and open symbols correspond to ${I}_{\mathrm{c}}^{\mathrm{P}(\mathrm{AP})}$ when pulse current (50 ns long) and DC current (1 s long) are used, respectively. Solid lines represent fitting a linear function to the data.

Figure 2 displays ${I}_{\mathrm{c}}^{\mathrm{P}(\mathrm{AP})}$ as a function of *H*_*y*_. The solid circles and triangles show ${I}_{\mathrm{c}}^{\mathrm{P}}$ and ${I}_{\mathrm{c}}^{\mathrm{AP}}$, respectively, using 50 ns long pulse current. We find ${|I}_{\mathrm{c}}^{\mathrm{P}(\mathrm{AP})}|$ increases when *H*_*y*_ increases the barrier height of the switching. For example, the lower energy state is the AP (P) state when *H*_*y*_ < *H*_s_ (*H*_*y*_ > *H*_s_). ${|I}_{\mathrm{c}}^{\mathrm{AP}}|$ is therefore larger than ${|I}_{\mathrm{c}}^{\mathrm{P}}|$ when *H*_*y*_ < *H*_s_. This is consistent with STT induced magnetization switching. The switching current ${I}_{\mathrm{c}}^{\mathrm{P}(\mathrm{AP})}$ when the pulse current is replaced with a DC current (duration 1 s) is shown by the open symbols in Fig. 2. We fit the data with a linear function to compare the slopes for pulse and DC current. The solid lines in Fig. 2 represent the least square fitting results. The estimated slopes are − 1.18 (solid triangles) and − 0.63 μA/Oe (solid circles) for the pulse current. In the case of DC current, we obtain − 0.44 (open triangles) and − 0.33 μA/Oe (open circles), which are smaller than those for the pulse current.

The inverse of the slope in Fig. 2 corresponds to the damping-like spin orbit effective field (${h}_{\mathrm{DL}}$) per unit current acting on the magnetization divided by the Gilbert damping constant $\alpha$ (see Methods). We find that the averaged ${h}_{\mathrm{DL}}/\alpha$ is ~  − 12.3 Oe (− 29.0 Oe) at a channel current density (j) of 10^6^ A/cm^2^ for pulse (DC) current. (The current density is estimated by assuming a uniform current flow within the channel.) We infer that difference in ${h}_{\mathrm{DL}}/\alpha$ estimated using the pulse and DC current measurements may be caused by difference in the switching regime (e.g. dynamical switching vs. thermally activated switching^17^). ${h}_{\mathrm{DL}}$ per current density is related to the effective spin Hall angle $\xi$ via ${h}_{\mathrm{DL} / j}\sim \hslash \xi /(2e{M}_{s}d)$, where ${M}_{s}$ and $d$ are the saturation magnetization and thickness of the free layer^4^. Substituting ${M}_{s}\cong 1200$ emu/cm^3^ and $\alpha \cong 0.04$ estimated in a similar system^12^ and $d=2$ nm, we find $\xi \cong -0.03 (-0.07)$ for the pulse (DC) current induced switching. We consider $\xi$ estimated from DC current induced switching is more accurate due to the smaller ${|I}_{\mathrm{c}}^{\mathrm{P}(\mathrm{AP})}|$; see Methods for the details. Note that $\xi$ is smaller than that estimated in previous works^10, 14, 18^ likely due to damages caused by device fabrication processes (e.g. Ar ion etching of the CoFeB free layer that needs to be stopped right above the W layer).

### Current and field dependence of the switching probability

The switching probability of the free layer is obtained using the following process: (1) Reset the free layer magnetization direction to either P or AP state using *H*_*y*_ ~ $\pm$ 1000 Oe. (2) Measure the MTJ resistance at *H*_*y*_ ~ 0. (3) Apply a bias field *H*_*y*_. For field-induced switching, we vary the amplitude and length of the bias field. To study current-induced switching, a pulse or DC current with variable amplitude is applied subsequently. (4) Measure the MTJ resistance at *H*_*y*_ ~ 0. The difference of the MTJ resistance acquired in processes (2) and (4) provides information on the magnetic state of the free layer. Processes (1)-(4) are repeated 20–50 times to obtain the switching probability.

**Figure 3:**
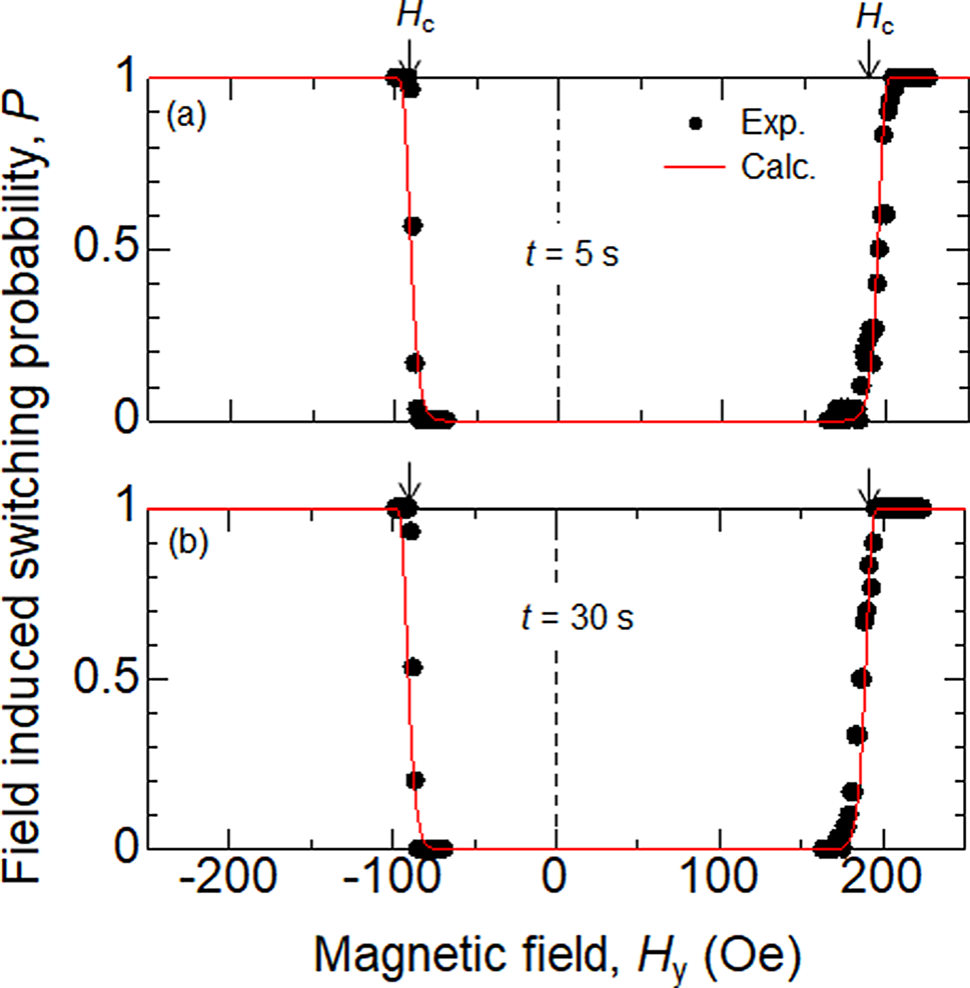
(**a**, **b**) Magnetization switching probability plotted as a function of magnetic field (*H*_*y*_). The duration of the field is 5 s (**a**) and 30 s (**b**). Symbols represent experimental data, the red solid lines show the fitting results using Eq. (1). The parameters used are: ${H}_{\mathrm{K}}:300$ Oe, ${H}_{\mathrm{s}}:54$ Oe, ${\Delta }_{\mathrm{P}}={\Delta }_{\mathrm{AP}}:85$ and $I=0$. The arrows represent *H*_c_ obtained in Fig. 1(b).

**Figure 4:**
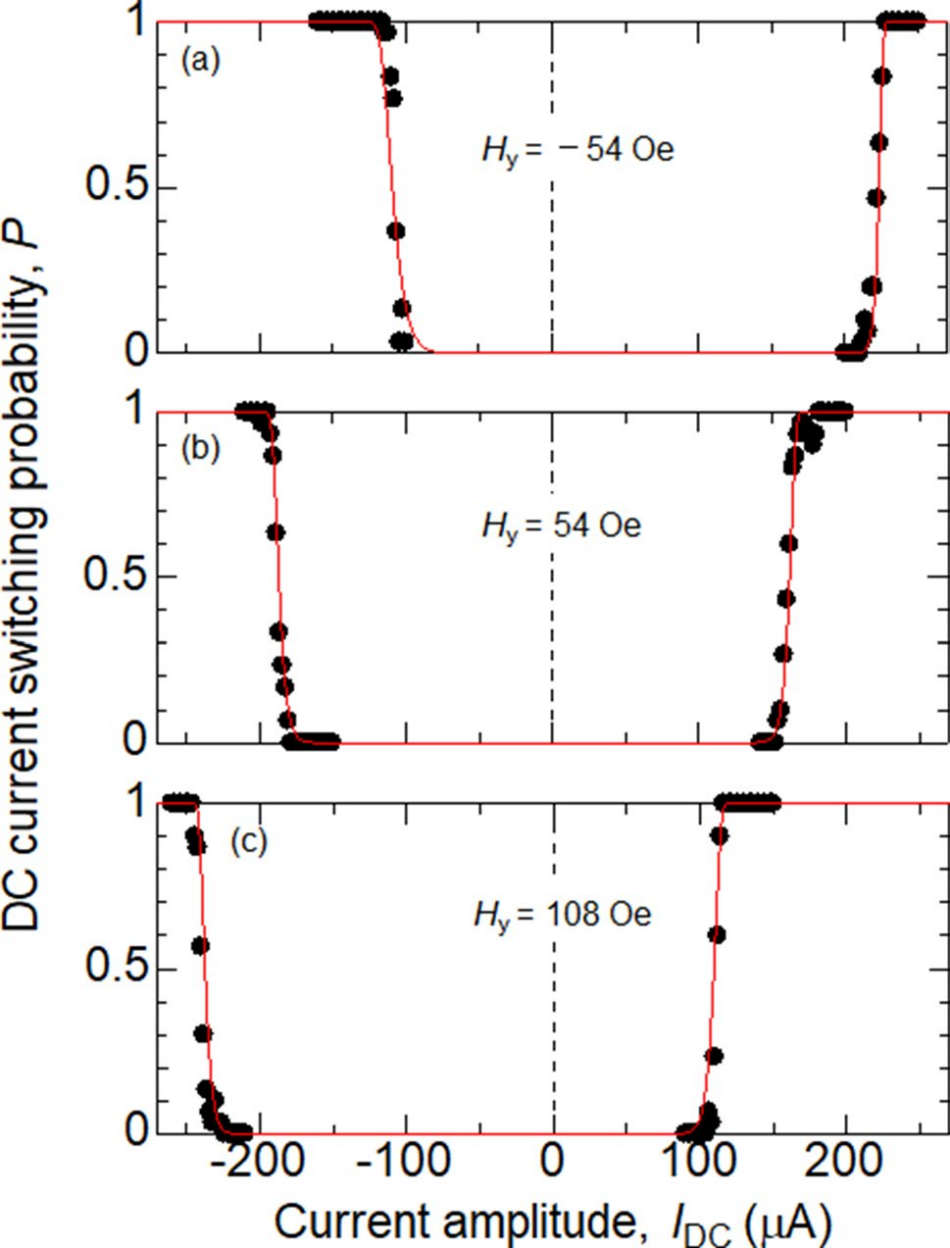
(**a**–**c**) Magnetization switching probability plotted as a function of the DC current *I*_DC_. The bias field *H*_*y*_ is varied as indicated. Switching probability is obtained using 1 s long DC current. Symbols represent experimental data, the red solid lines show the fitting results using Eq. (1). The parameters used for the fitting are listed in Table 1.

**Figure 5:**
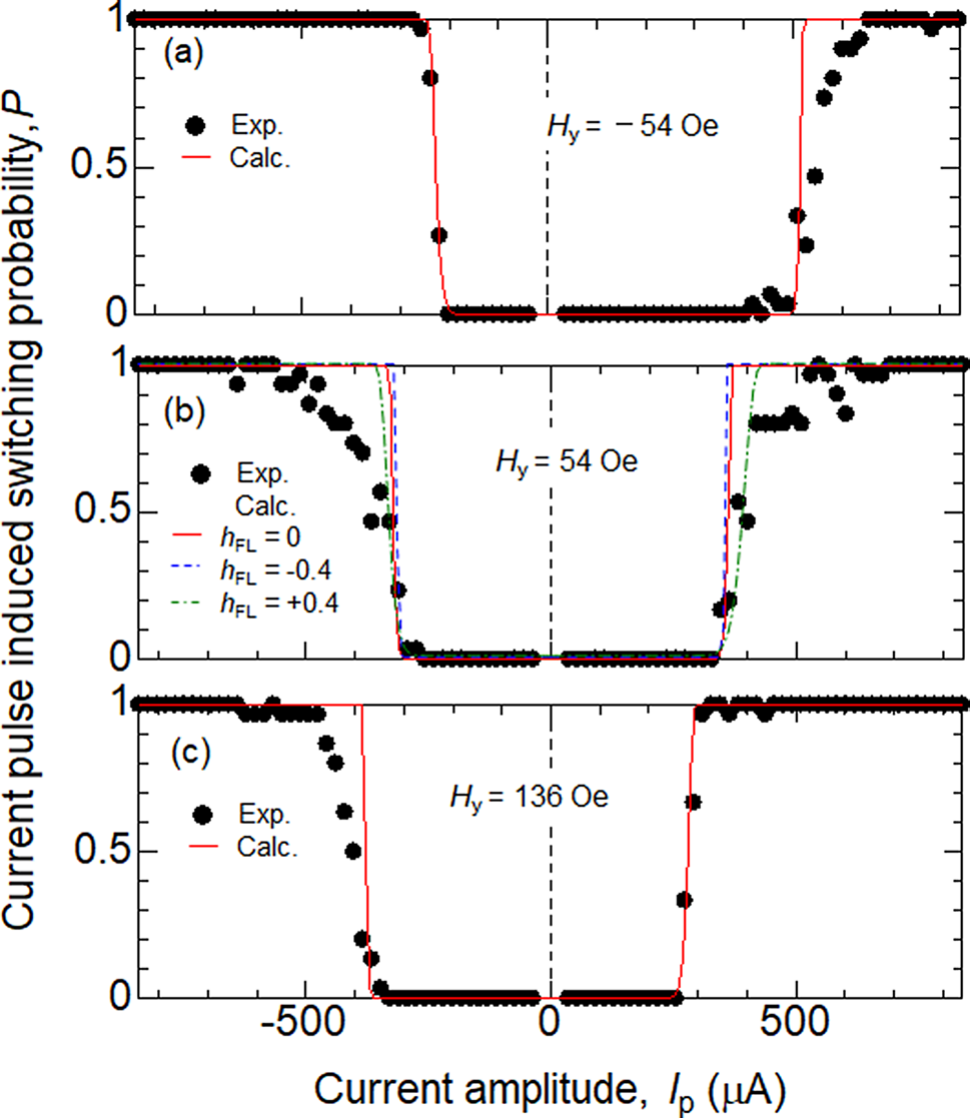
(**a**–**c**) Magnetization switching probability plotted as a function of the pulse current amplitude *I*_P_. The bias field *H*_*y*_ is varied as indicated. Switching probability is obtained using 50 ns long pulse current. Symbols represent experimental data, the red solid, blue dashed and green dotted lines show the switching probability calculated using Eq. (1) with different ${h}_{\mathrm{FL}}$. The parameters used for the fitting are listed in Table 2.

Figure 3a,b show the probability of field-driven magnetization switching plotted as a function of *H*_*y*_. The duration of *H*_*y*_ in process (3), denoted as *t*, is varied. The switching probability changes from 0 to 1 as the magnitude of *H*_*y*_ is increased. The transition of the switching probability from 0 to 1 is relatively sharp and shows little dependence on *t*. The switching characteristics of DC and pulse current induced magnetization switching are shown in Figs. 4 and 5, respectively. The duration of the pulse current is fixed to 50 ns (DC current is applied for ~ 1 s) and the bias field *H*_*y*_ is varied. Note that *H*_*y*_ < *H*_s_ (*H*_*y*_ > *H*_s_) favors the AP (P) state (*H*_s_ ~ 54 Oe). The transition of the switching probability, from 0 to 1, is sharp and nearly independent of *H*_*y*_ for the DC current induced switching. For *H*_*y*_ ~ *H*_s_, the transition shows a slight broadening when *P* approaches 1, a feature that is enhanced for pulse current induced switching.

In contrast, the transition shows a strong dependence on *H*_*y*_ for pulse current induced switching. First, for *H*_*y*_ ~ *H*_s_, we find a tail in the transition from 0 to 1 near *P* ~ 1, which was also apparent in the DC current switching. Although one may infer that such reduction in the switching probability at near zero (net) magnetic field is associated with magnetization switching processes that involve motion of domain walls, we do not find evidence of intermediate resistance states that correspond to domain walls remaining in the element after application of the current pulse. In addition, we find the transition width for which *P* varies from 0 to 1 depends on *H*_*y*_. The transition width tends to increase when ${H}_{y}$ is varied in a way to increase the barrier height for switching. This is not in line with the conventional view of thermally activated magnetization switching.

To show this discrepancy, the switching probability is calculated using a model based on thermally activated magnetization switching, given by^19^

1 $$\begin{aligned} P & = 1 - {\text{e}}^{ - \nu t} , \\ \nu & = \frac{1}{{\tau_{0} }}{\text{exp}}\left\{ { - \Delta_{{{\text{AP}}}} \left( {1 - \frac{{H_{y} - H_{{\text{S}}} + h_{{{\text{FL}}}} I}}{{H_{{\text{K}}} }}} \right)^{2} \left( {1 - \frac{I}{{I_{{\text{C}}}^{{{\text{AP}}}} }}} \right)} \right\} \quad {\text{for AP}} \to {\text{P}},{ } \\ \nu & = \frac{1}{{\tau_{0} }}{\text{exp}}\left\{ { - \Delta_{{\text{P}}} \left( {1 + \frac{{H_{y} - H_{{\text{S}}} + h_{{{\text{FL}}}} I}}{{H_{{\text{K}}} }}} \right)^{2} \left( {1 - \frac{I}{{I_{{\text{C}}}^{{\text{P}}} }}} \right)} \right\} \quad {\text{for P}} \to {\text{AP}} , \\ \end{aligned}$$

where ${\tau }_{0}$, $t$, and ${H}_{\mathrm{K}}$ represent the inverse of the attempt frequency, the duration of the driving force (current or bias field ${H}_{y}$), and the in-plane magnetic shape anisotropy field, respectively. ${\tau }_{0}$ is fixed to 1 ns here for simplicity. ${\Delta }_{\mathrm{P}(\mathrm{AP})}$ and ${I}_{\mathrm{C}}^{\mathrm{P}(\mathrm{AP})}$ are, respectively, the thermal stability factor and the switching current when the initial state is the P (AP) state. *I* is the current passed along the W channel: we substitute *I*_p_ and *I*_DC_ into *I* when pulse current and DC current, respectively, are applied. We also add an effective field that arises due to the field-like SOT (${h}_{\mathrm{FL}}$) that scales with $I$. The direction of the fields (${H}_{y}, {H}_{\mathrm{S}}, {h}_{\mathrm{FL}}$) and current are in accordance with the definitions shown in Fig. 1.

First, we study the switching characteristics of field induced magnetization switching (Fig. 3). We fit the data with Eq. (1) using ${\Delta }_{\mathrm{P}(\mathrm{AP})}$ and *H*_K_ as the fitting parameters and set $I=0$. The results, shown by the red solid lines in Fig. 3, are in good agreement with the data. We obtain ${\Delta }_{\mathrm{P}}\sim {\Delta }_{\mathrm{AP}}\sim 85$ and *H*_K_ ~ 300 Oe for all data measured. The size of *H*_K_ is in good agreement with the shape anisotropy field: from the dimension of the ellipse (370 nm $\times$ 120 nm $\times$ 2 nm) and the saturation magnetization of CoFeB (*M*_s_ ~ 1200 emu/cm^3^), *H*_K_ ~ 396 Oe. The difference of ${\Delta }_{\mathrm{P}}$ and ${\Delta }_{\mathrm{AP}}$ is negligible.

Next, we fit the data from current induced magnetization switching using Eq. (1) with *H*_K_ = 300 Oe, ${\Delta }_{\mathrm{P}(\mathrm{AP})}$= 85 (obtained from the results shown in Fig. 2) and *H*_s_ = 54 Oe. ${I}_{\mathrm{c}}^{\mathrm{P}(\mathrm{AP})}$ and ${h}_{\mathrm{FL}}$ are used as the fitting parameters. The fitted curves for DC current induced switching are shown by solid lines in Fig. 4a–c. The fitted curves show good agreement with the data. The slight broadening of transition near *P* ~ 1 for ${|H}_{y}-{H}_{\mathrm{S}}|$ ~ 0, however, cannot be reproduced. We note that ${h}_{\mathrm{FL}}$ does not significantly influence the fitting since ${h}_{\mathrm{FL}}I$ is small compared to ${H}_{y}-{H}_{\mathrm{S}}$. Parameters that provide best fit to the data are summarized in Table 1.

**Table 1:** Parameters used to fit the experimental data on DC current induced switching. The other fixed parameters are: ${H}_{\mathrm{K}}:300$ Oe, ${H}_{\mathrm{s}}:54$ Oe.

*Hy* (Oe)		Δ_P_	Δ_AP_	*I*_C_^P^ (μA)	*I*_C_^AP^ (μA)	*h*_FL_ (Oe/μA)
(a)	− 54	85	85	− 250	265	− 0.1
(b)	54	85	85	− 260	230	− 0.1
(c)	108	85	85	− 310	190	− 0.1

The fitting results of pulse current induced switching are shown in Fig. 5a–c. The parameters used in the calculations are summarized in Table 2. We find that the *H*_*y*_ dependence of the transition width cannot be accounted for by even when the field-like SOT is introduced. The solid, dashed and dotted lines in Fig. 5b show the switching probability calculated using Eq. (1) with different ${h}_{\mathrm{FL}}$. Since ${h}_{\mathrm{FL}}I$ scales with current, its effect on *P* is different from the external field ${H}_{y}$. We find that positive ${h}_{\mathrm{FL}}$ tends to broaden the transition. Note, however, that the sign of the field-like SOT in similar heterostructures have been found to be either negative or relatively small^20^. Moreover, the model calculations show significant deviation from the data, in particular, when $|{H}_{y}-{H}_{\mathrm{S}}|$ ~ 0. This is largely due to the broadening of transition near *P* ~ 1 for $|{H}_{y}-{H}_{\mathrm{S}}|$ ~ 0, which is larger than that of DC current induced switching.

**Table 2:** Parameters used to fit the experimental data on pulse current induced switching. The other fixed parameters are: ${H}_{\mathrm{K}}:300$ Oe, ${H}_{\mathrm{s}}:54$ Oe.

*Hy* (Oe)		Δ_P_	Δ_AP_	*I*_C_^P^ (μA)	*I*_C_^AP^ (μA)	*h*_FL_ (Oe/μA)
(a)	− 54	85	85	− 270	550	0
(b)	54	85	85	− 320	365	− 0.4
				− 340	380	0
				− 400	500	0.4
(c)	136	85	85	− 390	330	0

We briefly comment on the reason behind the change in the transition width with respect to ${h}_{\mathrm{FL}}$ in the model calculations (i.e. Equation (1)). First, ${h}_{\mathrm{FL}}$ influences ${I}_{\mathrm{c}}^{\mathrm{P}(\mathrm{AP})}$. With the current setting (when current is passed along $+x$, electrons with spin polarization along $+y$ diffuses in from the W layer to the CoFeB layer), positive ${h}_{\mathrm{FL}}$ increases ${|I}_{\mathrm{c}}^{\mathrm{P}(\mathrm{AP})}|$ (negative ${h}_{\mathrm{FL}}$ decreases ${|I}_{\mathrm{c}}^{\mathrm{P}(\mathrm{AP})}|$)^21, 22^. This is evident from the plots shown in Fig. 5b. Increase in ${|I}_{\mathrm{c}}^{\mathrm{P}(\mathrm{AP})}|$ indicates larger barrier height for thermally activated switching. In general, larger barrier height leads to smaller transition width for such switching process. Here, however, the field-like SOT scales with the current. Therefore, increase in current leads to further increase in the barrier height via the field-like SOT, which causes the transition broadening. Thus positive ${h}_{\mathrm{FL}}$ tends to increase the transition width.

## Discussion

Finally, we discuss possible mechanisms that cause the anomalous ${H}_{y}$ dependence of the transition width of the pulse current induced magnetization switching probability. First, it is possible that incoherent magnetization switching that involves nucleation and subsequent motion of domain walls can cause such broadening of the switching probability. However, we do not find intermediate resistance states (after application of current pulses) that suggest presence of domain walls in the free layer. The broadening is thus not caused by simple domain wall pinning effects. Current induced Joule heating may also play role in the switching process. For this purpose, we analyzed the data using a modified Eq. (1) that takes into account Joule heating. Here we assumed the barrier height (${\Delta }_{\mathrm{AP}(\mathrm{P})}$) decreases with increasing current: ${\Delta }_{\mathrm{AP}(\mathrm{P})}$ in Eq. (1) is replaced by $\frac{{\Delta }_{\mathrm{AP}(\mathrm{P})}}{1+{aI}^{2}}$, where $a$ is a fitting constant. We find that such modification cannot describe the ${H}_{y}$ dependence of the transition width of the pulse current induced switching. The nonuniformity of the stray field from the SAF pinned layer, if it were to play a role, will influence the switching probability of both pulse and DC current induced switching. Since the transition width is significantly larger for the pulse current induced switching, we consider its effect is negligible. We infer that the current amplitude required to induce switching plays a role^23, 24^. If the current amplitude exceeds a threshold above which the so-called dynamic switching occurs (thermally activated switching takes place below the threshold current), the transition width cannot be accounted for using Eq. (1) which is based on thermally activated switching. Further studies are required to clarify the mechanism of the transition width broadening of the pulse current induced switching.

In summary, we have studied SOT switching of a three terminal MTJ that consists of W channel, a CoFeB free layer, MgO barrier and a CoFeB synthetic antiferromagnetic pinned layer. The probability of current induced magnetization switching, as a function of easy axis bias magnetic field, are evaluated and compared to calculations based on thermally activated switching. From the dependence of the switching current on the bias magnetic field, the effective spin Hall angle is estimated to be − 0.07. We find the model can account for the field-induced switching and, to a lesser degree, DC current induced switching. In contrast, the change in the switching probability with pulse current cannot be described even when contribution from the field-like torque is included. We infer that the current amplitude that determines the switching mode (dynamical switching vs. thermally activated switching) plays a role.

## Methods

The damping-like spin orbit effective field (${h}_{\mathrm{DL}}$) relates to the effective spin Hall angle $\theta$ via

2 $${h}_{\mathrm{DL}}=\frac{\hslash \theta j}{2e{M}_{s}d},$$

where $j$ is the current density flowing in the W channel along the $x$ direction, ${M}_{s}$ and $d$ are the saturation magnetization and thickness of the free layer. The critical current density of in-plane magnetized free layer at zero temperature is given by^25^

3 $${j}_{\mathrm{c}}=\frac{2\alpha e{M}_{s}d}{\hslash \theta }\left({H}_{y}+{H}_{\mathrm{K}}+\frac{{H}_{\mathrm{demag}}}{2}\right),$$

where $$\alpha$$, $${H}_{\mathrm{K}}$$, and $${H}_{\mathrm{demag}}$$ are the Gilbert damping constant, in-plane magnetic anisotropy field, and perpendicular magnetic anisotropy field, respectively. The in-plane magnetic anisotropy field originates from the shape anisotropy^12^, and is primarily determined by the demagnetization coefficients $${N}_{i}$$ ($$i=x,y,z)$$ via $${H}_{\mathrm{K}}=4\pi M\left({N}_{x}-{N}_{y}\right)$$. The perpendicular magnetic anisotropy field is given by $${H}_{\mathrm{demag}}=4\pi M\left({N}_{z}-{N}_{x}\right)-{H}_{\mathrm{K}\perp }$$, where $${H}_{\mathrm{K}\perp }$$ represents the interfacial perpendicular magnetic anisotropy field^26–28^. Equations (1) and (3) are commonly used to analyze the probability of spin-transfer torque switching^29^, where it is often assumed that the effective switching barrier height linearly depends on the current when the current pulse-width is sufficiently long^30–32^. When the pulse width of the current is narrow, a large current will be necessary to induce a fast switching. A large current suppresses the effective switching barrier significantly, and as a result, the high energy-barrier assumption^33^ used in the derivation of Eq. (1) becomes no longer applicable. Therefore, the DC current induced switching measurement provides more accurate value of the effective spin Hall angle compared to that estimated by using the pulse current. From Eqs. (2) and (3), $${h}_{\mathrm{DL}}$$ per current density can be estimated as $${\mathrm{d}j}_{\mathrm{c}}/\mathrm{d}{H}_{\mathrm{y}}=\alpha j/{h}_{\mathrm{DL}}$$.

## Data Availability

The data that support the findings of this study are available from the corresponding author upon reasonable request.
